# Spanish validation endorsement of SDM-Q-9, a new approach

**DOI:** 10.1186/s12889-019-6436-7

**Published:** 2019-01-23

**Authors:** Geovanny Efraín Alvarado-Villa, Jorge Daniel Moncayo-Rizzo, Jorge Andrés Gallardo-Rumbea

**Affiliations:** Universidad de Especialidades Espíritu Santo, Samborondón, Ecuador

**Keywords:** Shared decision making, SDM-Q-9 questionnaire, Confirmatory factor analysis, Psychometrics, Doctor-patient relationship, Diabetes mellitus

## Abstract

**Background:**

The Shared Decision Making (SDM) model allows the patient to be part of their own disease treatment and control. The translation to Spanish of a questionnaire that measures the patient perception of SDM will allow enlarging the range of its application. However, the essence of the questionnaire can be altered during its translation, which could curb the appreciation of the question and what the question originally asked for. The objective of this study is to evaluate the application of SDM-Q-9 in its psychometric properties, to a Spanish speaking population after its translation process.

**Method:**

The questionnaire was given to 76 outpatients who attended a medical control at the hospital. The informed consent process was developed before the patient underwent the physician’s evaluation, and the SDM-Q-9 was applied when the patient finished the medical evaluation. The reliability of the questionnaire was evaluated and its structural validity was verified by the exploratory factor analysis (EFA) and the confirmatory factor analysis (CFA).

**Results:**

The SDM-Q-9 presented reliability and validity according to the following indicators. The internal consistency, measured by Cronbach’s alpha, was 0.839 for the whole scale. The EFA showed a bi-dimensional solution, but the CFA indicated that the model with best indices of fit was the one-dimensional solution, excluding the first item. The indices used where: CFI 0.953, RMSEA (IC) 0.076 (0.000–0.134) for model 2, and CFI 0.961, RMSEA 0.071 (0.000–0.132) for model 5 are better.

**Conclusion:**

The questionnaire adaptation to the Latin American Spanish language has displayed reliability and validity according to the Cronbach’s alpha indicators.

## Background

In recent years, the role of patients in the selection of their treatment has evolved. Patients are no longer considered the passive subject; instead, they have acquired a more active participatory roll in the decision making process. Shared decision-making (SDM) is a model where both physician and patient are included in the decision-making process of the treatment [1–3]; this contrasts the “paternalistic” model in which the doctors make the decision for the treatment according to their knowledge, experience and scientific basis without taking into account the preferences and values of the patient [1]. The SDM model focuses on promoting the value of the doctor-patient relationship in a way that the conversation between both parties could meet to discuss not only the pros and cons of the treatment options, but also the patient’s preferences; so both the patient and the doctor can agree on a specific treatment [2, 4–6].

The implementation of this model has been carried out in several hospitals and health care centers in developed countries, with great acceptance by doctors and patients [7, 8]. In addition, its use has been evaluated not only in primary care, but also in specialized care, such as for oncological [9] and neurological diseases [10]. However, the perspectives about the decision-making process differ, not between doctors and patients, but also among the patients themselves. This is because not all patients may be interested in taking on an active role in the decision-making process [11, 12]. Consequently, certain tools have been developed to measure the level of SDM in each person involved in the decision-making process.

The 9-item questionnaire (SDM-Q-9) is one of the instruments used by the patients to measure the level of SDM with their physicians during a consult. SDM-Q-9 originally written in the English language [1]. This questionnaire created by D. Simon, et al. was designed based on the shared decision-making questionnaire (SDM-Q) [13]. The SDM-Q-9 has been translated into many different languages, but very few have been validated. One of the validated versions was made by Carlos de las Cuevas in Spain [3]. The internal consistency of this Spanish version of the questionnaire was validated in a sample of 540 patients, with a Cronbach’s alpha of 0.885 for the entire scale [3]. The latest version validated by Álvarez [14], in Spanish, obtained 0.89 of Cronbach’s alpha for the entire scale with a sample of 239 patients [14].

Therefore, this study aims to propose a new SDM-Q-9 validated version in Spanish and evaluate its psychometric properties using a sample of patients with type 2 diabetes mellitus.

## Methods

### Instrument

Based on the SDM-Q created by D. Simón in 2006 [13], the SDM-Q-9 was designed and validated in English in 2010 [1]. This consists of nine questions that evaluate the steps of SDM. Each item is scored with a Likert scale of 6 options (from Totally Disagree to Fully Agree), so the sum of all items resolves between 0 and 45. This value is multiplied by 20/9 allowing a scale between 0 and 100, where 0 is the lowest possible SDM level and 100 is the highest possible SDM level.

### Translation

To employ the SDM-Q-9 in Ecuador, the questionnaire was translated into Spanish language and validated according to the international criteria proposed by Sperber, A.D. [15]. Two native speakers with fluent command of Spanish and English languages independently translated the questionnaire to Spanish language. Both translations were compared and a version that gathers the most reliable translation for each question was developed. The Spanish version was translated back into English by two other translators with the academic title of translator and interpreter. Again, the translations were compared and a version that includes the most reliable translation for each question was developed. Translators do not know the concept and purpose of the translated material; those who perform the translation from Spanish to English have no knowledge about the original questionnaire.

To obtain the translated questionnaire, the original version of the SDM-Q-9 questionnaire was compared with the English translation. The comparison was made by 30 bilingual students who study degrees completely in English. Each question of each questionnaire was classified by two criteria: Criterion (A) is based on the comparability of the language, and criterion (B) on the similarity of the interpretability. Each criterion has a score scale from 1 (extremely comparable / extremely similar), 4 (moderately comparable/ moderately similar) to 7 (nothing comparable / nothing similar) according to the Sperber, A.D. [15]. Next, the average was calculated for each question. The previously translated Spanish version of the questions with average less than or equal to 3 can be used to be part of the final version of the questionnaire. On the other hand, any question with an average higher than 3 requires a litany of the entire translation and assessment process by the 30 bilingual students until the question obtains an average score less than or equal to 3.

This process allowed us to identify questions with dubious translation and thus retranslate them until they are interpreted equally in both languages. After making the necessary adjustments and transcultural adaptations, the Spanish version of the SDM-Q-9 questionnaire was ready for use.

### Sample and procedure

For the sample size calculation, a Fisher transformation for the Pearson correlation coefficient was used, with a statistical power 1 – β, and work safety of 1- $\frac{\alpha }{2}$, for a desired correlation magnitude (r) to be detected. Applying the formulas for the sample calculation of a correlational study, with a bilateral approach, a 95% safety and an 80% statistical power, a sample of 85 participants was obtained. This goes according to the sample size suggested for a factorial analysis, which is calculated multiplying the number of parameters or items by 5–20 [16]. Considering the SDM-Q-9 has 9 items or parameters, the sample range is 45–180 participants.

This study was carried out in two phases of work. In the first phase, between September and October 2017, 107 adult patients (over 18 years of age) with poorly controlled type 2 diabetes mellitus (glycosylated hemoglobin (HbA1c) ≥7%) that attended the outpatient office of Teodoro Maldonado Carbo Hospital in Guayaquil were invited to participate in the study. Each of the participants was invited before entering their usual medical consult. Those patients who expressed interest in participating began the informed consent process.

To optimize time, a sociodemographic questionnaire (sex, age and level of education) was done prior to entering the consult, as well as anthropometric measurements (weight, height, body mass index (BMI)), and if they visit a private doctor. After the consult and without the presence of the attending physician, the SDM-Q-9 was performed as a face-to-face interview, so each patient gave feedback about the recent consult as a reference to complete the questionnaire. Finally, the patients were taken to a certified laboratory where they underwent an HbA1c test. In the second phase, patients were contacted three months after the laboratory examination to perform a second HbA1c test and a survey regarding the control of their disease.

### Data analysis

Descriptive statistics were used for demographic data such as age, sex and level of education, as well as anthropometric measurements (weight, height and BMI calculation). Normality tests were performed on the different variables. For variables with normal distribution, the Pearson correlation test was used and for the non-normal distribution variables the Spearman correlation test was used. The analysis of the items included the mean and standard deviation of each item. The internal consistency and reliability analysis of the scale was performed with the α-Cronbach.

The study also used methods of exploratory factor analysis (EFA) to obtain the dimensionality of the scale. The method used was principal component analysis (PCA) with Varimax rotation. To determine if the items are sufficiently interrelated, the Bartlett sphericity test and the sample adequacy measure of Kaiser-Mayer-Olkin (KMO) were used. In addition, the confirmatory factor analysis (CFA) was used in order to demonstrate the factorial structural validity and consequent validity of inferred theoretical deductions.

Other tests used were the indices for goodness of fit: the chi-square, the comparative adjustment index (CFI), the roots mean square error of approximation (RMSEA). And the parsimony indexes: the Akaike information criterion (AIC), Bayesian information criterion (BIC) and Consistent AIC (CAIC). The criteria to measure the fit of the models obtained by the CFA, according to Hu and Bentler [17], were: CFI >0.9 and RMSEA < 0.08. And the lower value of AIC, BIC and CAIC of the models, comparing the adjustment of each of these [18, 19]. For these analyses, we used the SPSS program version 24 for Windows with the AMOS 22 application.

## Results

One hundred and seven patients were invited to participate in the study, of which 76 (71.03%) of them met the approved inclusion criteria and completed the phases of the study. All of the participants answered the 9 questions of the SDM-Q-9. Table 1 shows the distribution of the demographic variables. Although most of the participants were women (57.9%), this data does not produce any proportion bias (*p* = 0.207). The age range was between 35 and 86 years old, with an average of 60.42 years (SD = 9.585); the most frequent age group was 50–64 years old. Regarding the level of education, 1.3% had no education, 25% had primary education, 42.1% had secondary education and 31.6% had a university degree.

**Table 1:** Demographic Data Results

		N (76)	%	*p* Value
SEX	FEMALE	44	57.9	0.207
	MALE	32	42.1	
AGE GROUP	35–49	10	13.2	< 0.001
	50–64	46	60.5	
	65+	20	26.3	
EDUCATION LEVEL	NO STUDIES	1	1.3	< 0.001
	PRIMARY	19	25.0	
	SECONDARY	32	42.1	
	CERTIFICATE OF HIGHER EDUCATION	24	31.6	
	GRADUATE CERTIFICATE	0	0.0	

Table 2 shows the mean, standard deviation and the total correlation of items corrected by each question of the questionnaire. The range of the mean of the items is from 3 (item 6) to 4.88 (item 1). The first item obtained a low total correlation of items corrected (r = 0.360) in relation to the rest (0.474–7.34). Cronbach’s alpha for the whole scale is 0.839, and 0.841 excluding the first item.

**Table 2:** Statistical analysis validation of the SDM-Q9

	Mean (DS)	Corrected Item-Total Correlation	Cronbach’s Alpha if Item Deleted
SDM QUESTION 1	4.88 (1.833)	.360	.841
SDM QUESTION 2	3.80 (2.046)	.598	.817
SDM QUESTION 3	3.11 (2.213)	.474	.831
SDM QUESTION 4	3.70 (2.257)	.542	.824
SDM QUESTION 5	4.82 (1.749)	.511	.827
SDM QUESTION 6	3.00 (2.026)	.599	.817
SDM QUESTION 7	3.62 (2.097)	.734	.801
SDM QUESTION 8	3.11 (2.260)	.523	.826
SDM QUESTION 9	4.45 (1.976)	.625	.815

The KMO value was 0.833 and the Bartlett sphericity test was significant (x^2^ = 220.260, df = 36, *p* < 0.001), indicating that the factor analysis of the data is appropriate. The PCA allowed obtaining 2 components. Prior to Varimax rotation, the two components delivered 44.519 and 11.963% of the variance, respectively. Items 3–6 and 9 have readings above 0.5 in the first component; on the other hand, items 1,2,7 and 8 have their highest readings in the second component.

**Fig. 1:**
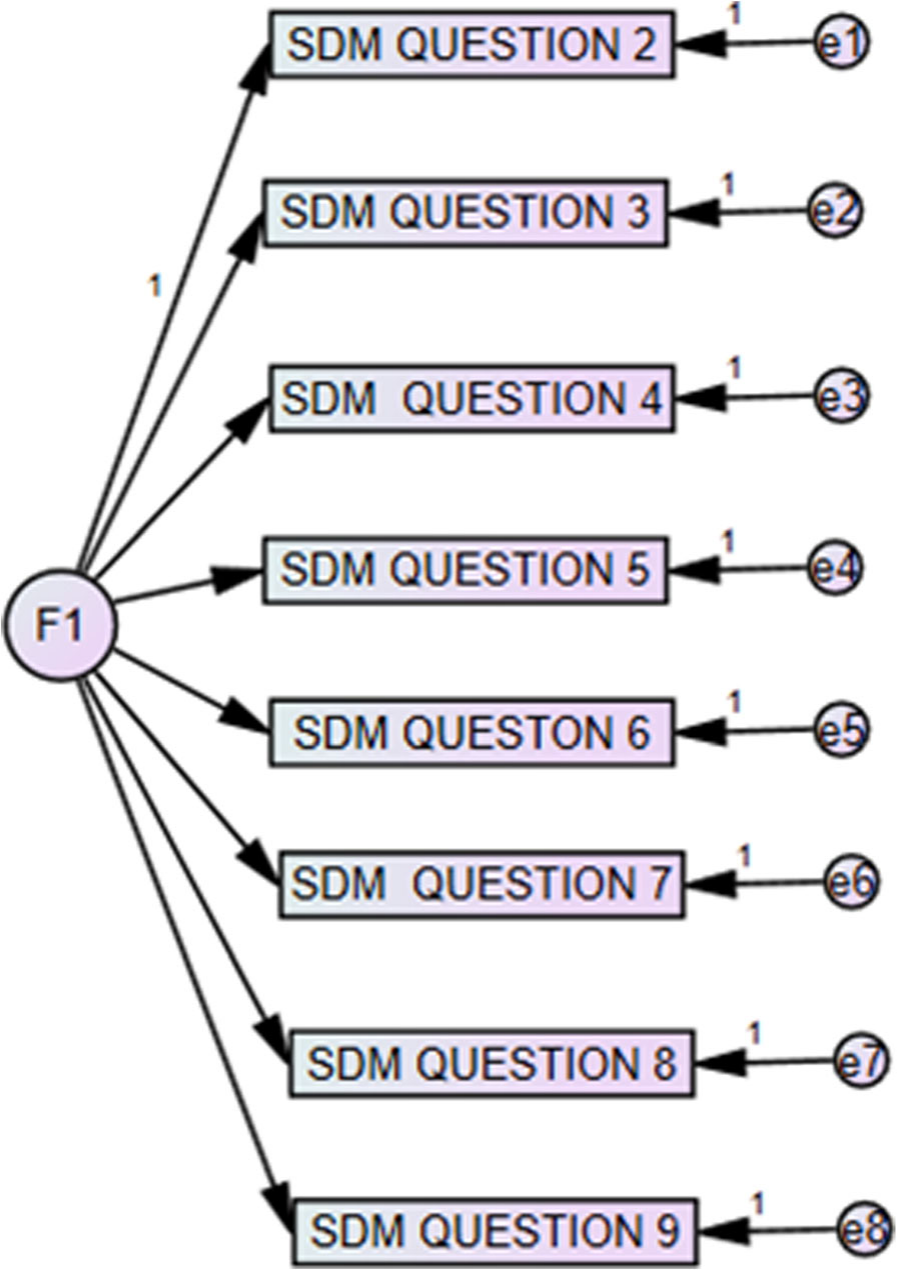
Model 2 – Unifactorial with exclusion of the first item

**Fig. 2:**
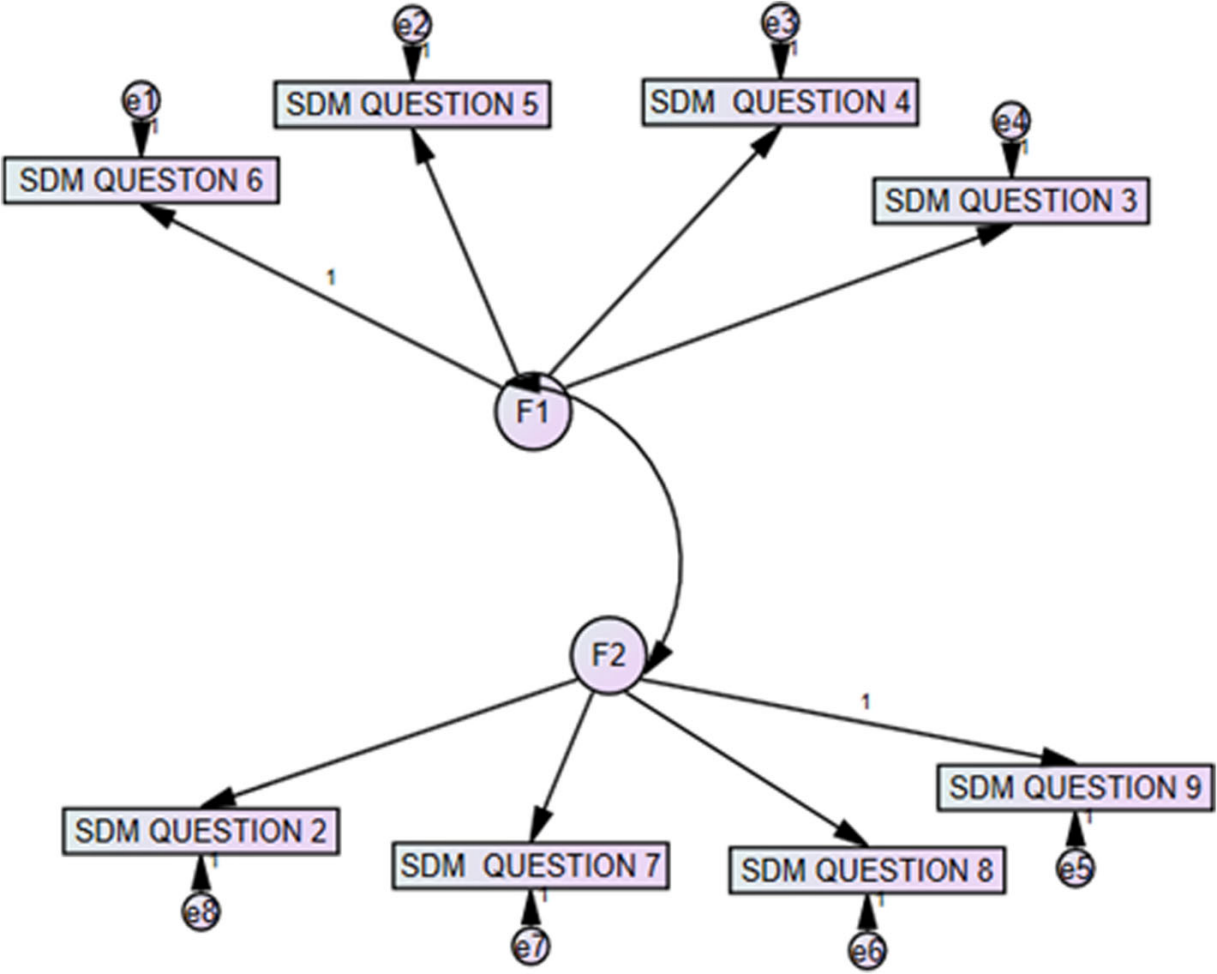
Model 5 – Bifactorial with exclusion of the first item

The CFA was performed in five models proposed according to the results of the PCA and considering models of the last validated versions of the questionnaire [3, 14] (Table 3). The original unifactorial model (Model 1); the unifactorial model excluding the first item (Model 2) suggested by different authors [3, 14] (see Fig. 1); and three bifactorial models obtained by the PCA. Model 3 includes, in the first factor, items 3–6, 9 and in the second factor, items 1, 2, 7, 8. Model 4 corresponds to the first factor with items 3–6 and the second factor with Items 1, 2, 7–9. Finally, Model 5, excluding the first item, contains items 3–6 in the first factor, and items 2, 7, 8, 9 in the second factor (see Fig. 2). The latter, together with Model 2, obtained the best indices of adjustment (CFI, RMSEA, AIC, BIC and CAIC).

**Table 3:** Results of the confirmatory factor analysis (CFA)

	X^2^	p value	CFI	RMSEA (IC)	AIC	BIC	CAIC
Model 1	40.122	0.050	0.933	0.080 (0.02–0.130)	76.122	118.075	136.075
Model 2	28.595	0.096	0.953	0.076 (0.000–0.134)	60.595	97.887	113.887
Model 3	38.385	0.056	0.937	0.080 (0.000–0.130)	76.385	120.669	139.669
Model 4	36.897	0.076	0.944	0.075 (0.000–0.126)	74.897	119.181	138.181
Model 5	26.189	0.125	0.961	0.071 (0.000–0.132)	60.189	99.811	116.811

## Discussion

The present study reports the psychometric properties of the new Spanish version of the SDM-Q-9 in a sample of patients with poorly controlled type 2 diabetes mellitus. The translation and validation of SDM-Q-9 has been carried out according to the guide for cross-cultural investigations [15].

The new Spanish version of the SDM-Q-9 has shown reliability and factorial validity. The internal consistency, determined with the α-Cronbach coefficient for the whole scale, is considered adequate for the study (α-Cronbach = 0.839). This result can be compared with other studies done such as Álvarez et al. [14] and De Las Cuevas et al. [3], where this index of reliability was 0.89. As our sample is smaller in comparison to those studies, the result obtained is considered adequate for the reliability of the instrument.

The total correlation of the corrected item was between 0.474 and 0.734, with the exception of the first item that has a lower value (r = 0.360). These results are comparable with other authors such as Álvarez et al. [14], De Las Cuevas et al. [3], and Kriston et al. [1], where the first item had a lower value compared to the rest; these were respectively: 0.45–0.88 [14], 0.272–0.820 [3] and 0.685–0.826 [1].

The PCA allowed obtaining two bifactorial models: one of them with the nine items (Model 3) and the other excluding the first item (Model 5). The obtained model contains, in the first factor, items 3–6 and 9 and, in the second factor, items 1, 2, 7 and 8 (Model 3). The following model (Model 5) contains in the first factor items 3–6, and in the second factor items 2, 7, 8 and 9. However, another bifactorial model was proposed according to the theoretical conception of the questions, where the first factor comprised items 3–6 and the second factor items 1, 2, 7, 8 and 9 (Model 4). On the other hand, the original version showed a unifactorial structure [1] (Model 1), in addition to the most recent validations that suggested a unifactorial model excluding the first factor [3, 14] (Model 2), so it was decided to test the hypotheses. The CFA showed that models 2 and 5, unifactorial and bifactorial respectively, links better to the observed variables.

The correlation between the items follows the line of the rest of the studies, where the first item presents correlation problems. This means that the Cronbach’s alpha improves with the exclusion of the first item (from 0.835 to 0.841), so that the adjustment indices are better with the models in which the first item is excluded (Model 2 and 5 vs. Model 1, 3 and 4).

The adjustment index of model 2 (CFI = 0.953 and RMSEA = 0.076) and of model 5 (CFI = 0.961 and RMSEA = 0.071) presented better values ​​with respect to those obtained by Álvarez et al. [14] (CFI = 0.821 and RMSEA = 0.092). The possible reason for this is the level of translation presented by the questionnaire, as well as cultural reasons. Establishing the questions as “My doctor” instead of “My provider”, as was done by Álvarez et al. [14], allows greater understanding of the questions by patients.

Finally, when comparing the parsimony index of models 2 (IAC = 60,595, BIC = 97,887 and CAIC = 113,887) and 5 (IAC = 60,189, BIC = 99,811 and CAIC = 116,811) we obtain that model 2, unifactorial excluding the first item, presents better fit to the data. These results are similar to those obtained by other authors, where they suggest that the best model is unifactorial, but not bifactorial [3, 14].

This study has different limitations. The most relevant is the size of the sample (76 patients), so this sample cannot be representative of the population that has poorly controlled type 2 diabetes mellitus. Another limitation implies the use of the instrument only in patients with diabetes. Future research can apply the instrument to different diseases such as cancer, cardiovascular diseases, or different diseases of chronic and/or acute type where there is a wide variety of treatments, in which the shared decision-making model could improve the attachment and satisfaction of the patients for the treatment.

## Conclusions

The questionnaire’s adaptation to the Latin American Spanish language has displayed reliability and validity according to the Cronbach’s alpha indicators. The results indicate that the new adaptation in Spanish of the SDM-Q-9 is suitable to be used in future research about SDM in Ecuador and other Spanish-speaking countries. The dimensionality of the instrument is maintained as a measure as previously corroborated by the authors. In the case of the indicators of variation and multidimensionality models, they were shown to be superior to the previous ones.

Because of its lack of internal consistency, question 1 had to be taken out from the model. Previously, this question had already generated dilemma in results and also translation. When removing this question, the questionnaire shows cohesion.

As this questionnaire is valid and reliable for Latin American culture, it should be used as a method to measure the level of SDM in health centers in different medical areas, allowing the development and application of strategies to encourage the use of the SDM model by treating physicians. Finally, the SDM-Q-9 will allow us to make a relation between the level of shared decisions and the glycosylated hemoglobin changes in the sample of patients with type 2 diabetes mellitus.

## Abbreviations

AIC: Akaike information criterion
BIC: Bayesian information criterion
BMI: Body-mass-index
CAIC: Consistent AIC
CFA: Confirmatory factor analysis
CFI: Comparative adjustment index
EFA: Exploratory factor analysis
HbA1c: glycosylated hemoglobin
KMO: Kaiser-Mayer-Olkin
PCA: Principal component analysis
RMSEA: Roots mean square error of approximation
SDM: Shared Decision-Making
SDM-Q: Shared decision-making questionnaire
SDM-Q-9: Shared decision-making 9-item questionnaire

## References

[1] Kriston L, Scholl I, Hölzel L, Simon D, Loh A, Härter M (2010). The 9-item shared decision making questionnaire (SDM-Q-9). Development and psychometric properties in a primary care sample. Patient Educ Couns.

[2] Rodenburg-Vandenbussche S, Pieterse AH, Kroonenberg PM, Scholl I, van der Weijden T, Luyten GPM (2015). Dutch translation and psychometric testing of the 9-item shared decision making questionnaire (SDM-Q-9) and shared decision making questionnaire-physician version (SDM-Q-doc) in primary and secondary care. PLoS One.

[3] De las Cuevas C, Perestelo Perez L, Rivero Santana A, Cebolla Martí A, Scholl I, Härter M (2014). Validation of the Spanish version of the 9 item shared decision making questionnaire. Health Expect.

[4] Ciapponi A (2012). Toma de decisiones compartidas. Evid Actual En Práctica Ambulatoria.

[5] Corser W, Holmes-Rovner M, Lein C, Gossain V (2007). A shared decision-making primary care intervention for type 2 diabetes. Diabetes Educ.

[6] Murray E, Pollack L, White M, Lo B (2007). Clinical decision-making: physicians’ preferences and experiences. BMC Fam Pract.

[7] Elwyn G, Laitner S, Coulter A, Walker E, Watson P, Thomson R (2010). Implementing shared decision making in the NHS. BMJ.

[8] Coulter A (2010). Do patients want a choice and does it work?. BMJ.

[9] Calderon C, Jiménez-Fonseca P, Ferrando PJ, Jara C, Lorenzo-Seva U, Beato C, et al. Psychometric properties of the Shared Decision-Making Questionnaire (SDM-Q-9) in oncology practice. Int J Clin Health Psychol. 2018; [citado 28 de marzo de 2018]; Disponible en: http://www.sciencedirect.com/science/article/pii/S1697260018300012. 10.1016/j.ijchp.2017.12.001 PMC6225052 30487919

[10] Ballesteros J, Moral E, Brieva L, Ruiz-Beato E, Prefasi D, Maurino J (2017). Psychometric properties of the SDM-Q-9 questionnaire for shared decision-making in multiple sclerosis: item response theory modelling and confirmatory factor analysis. Health Qual Life Outcomes.

[11] Adrian E, Glyn E (2006). Inside the black box of shared decision making: distinguishing between the process of involvement and who makes the decision. Health Expect.

[12] Levinson W, Kao A, Kuby A, Thisted RA (2005). Not all patients want to participate in decision making. J Gen Intern Med.

[13] Simon D, Schorr G, Wirtz M, Vodermaier A, Caspari C, Neuner B (2006). Development and first validation of the shared decision-making questionnaire (SDM-Q). Patient Educ Couns.

[14] Alvarez K, Wang Y, Alegria M, Ault-Brutus A, Ramanayake N, Yeh Y-H (2016). Psychometrics of shared decision making and communication as patient centered measures for two language groups. Psychol Assess.

[15] Sperber AD. Translation and validation of study instruments for cross-cultural research. Gastroenterology. 126:S124–S128. 10.1053/j.gastro.2003.10.016 14978648

[16] Suhr DD. Exploratory or Confirmatory Factor Analysis? Cary, NC: SAS Institute; 2006. Internet resource http://www2.sas.com/proceedings/sugi31/200-31.pdf.

[17] Hu L, Bentler PM. Cutoff criteria for fit indexes in covariance structure analysis: Conventional criteria versus new alternatives. Structural Equation Modeling: A Multidisciplinary Journal. 1999;6(1):1–55. 10.1080/10705519909540118.

[18] Caballero F. Selección de modelos mediante criterios de información en análisis factorial. Aspectos teóricos y computacionales. Universidad de Granada; 2011 [citado 2 de junio de 2018]. Available in: https://dialnet.unirioja.es/servlet/tesis?codigo=22590.

[19] Arredondo NHL, Rogers HL, Tang JFC, Gómez SLP, Arizal NLO, Pérez MÁJ (2012). Validación en Colombia del cuestionario MOS de apoyo social. Int J Psychol Res.

